# Hypermethylation of *DAPK1* is an independent prognostic factor predicting survival in diffuse large B-cell lymphoma

**DOI:** 10.18632/oncotarget.2394

**Published:** 2014-11-03

**Authors:** Lasse Sommer Kristensen, Fazila Asmar, Konstantinos Dimopoulos, Mette Kathrine Nygaard, Derya Aslan, Jakob Werner Hansen, Elisabeth Ralfkiaer, Kirsten Grønbæk

**Affiliations:** ^1^ Department of Haematology, Rigshospitalet, Blegdamsvej 9, 2100-DK, Copenhagen, Denmark; ^2^ Department of Haematology, Aalborg University Hospital, Mølleparkvej 4, 9000-DK, Aalborg, Denmark; ^3^ Department of Pathology, Rigshospitalet, Copenhagen, Denmark

**Keywords:** Diffuse Large B-Cell Lymphoma, Rituximab, prognostic markers, allele-specific DNA methylation, *DAPK*, *DAPK1*, mutations, *TP53*

## Abstract

Diffuse large B-cell lymphoma (DLBCL) is the most common type of non-Hodgkin's lymphoma. Improvements in overall survival have been observed with the introduction of rituximab in combination with cyclophosphamide, doxorubicin, vincristine, and prednisone (R-CHOP), however, prognostic markers are still needed. Methylation of the death associated protein kinase (*DAPK* or *DAPK1*) gene and *TP53* mutations are likely to have prognostic value in DLBCL. We have assessed *TP53* mutations and allelic *DAPK1* methylation patterns in a cohort of 119 DLBCL patients uniformly treated with R-CHOP-like regimens. We found that *DAPK1* promoter methylation was associated with shorter overall survival (*p*=0.017) and disease-specific survival (*p*=0.023). In multivariate analyses *DAPK1* methylation remained as an independent prognostic factor predicting disease-specific survival (*p*=0.038). When only considering individuals heterozygous for the rs13300553 SNP monoallelic methylation of the A-allele was associated with shorter overall- and disease-specific survival (*p*<0.001). Patients carrying both *DAPK1* methylation and a *TP53* mutation had an inferior survival compared to patients carrying only one of these molecular alterations, however, this was borderline statistically significant. Allele-specific *DAPK1* methylation patterns were also studied in a cohort of 67 multiple myeloma patients, and all of the methylated multiple myeloma samples heterozygous for the rs13300553 SNP were methylated on both alleles.

## INTRODUCTION

Improvements in overall survival of diffuse large B-cell lymphoma (DLBCL) patients have been observed with the introduction of rituximab in combination with cyclophosphamide, doxorubicin, vincristine, and prednisone (R-CHOP), however, prognostic markers are still needed [1]. Methylation of the death associated protein kinase (*DAPK* or *DAPK1*) gene and *TP53* mutations are likely to have prognostic value in DLBCL, and a better understanding of the molecular pathways leading to DLBCL progression may be important for the development of novel therapies aiming at causing DLBCL cells to undergo apoptosis [2].

**Figure 1:**
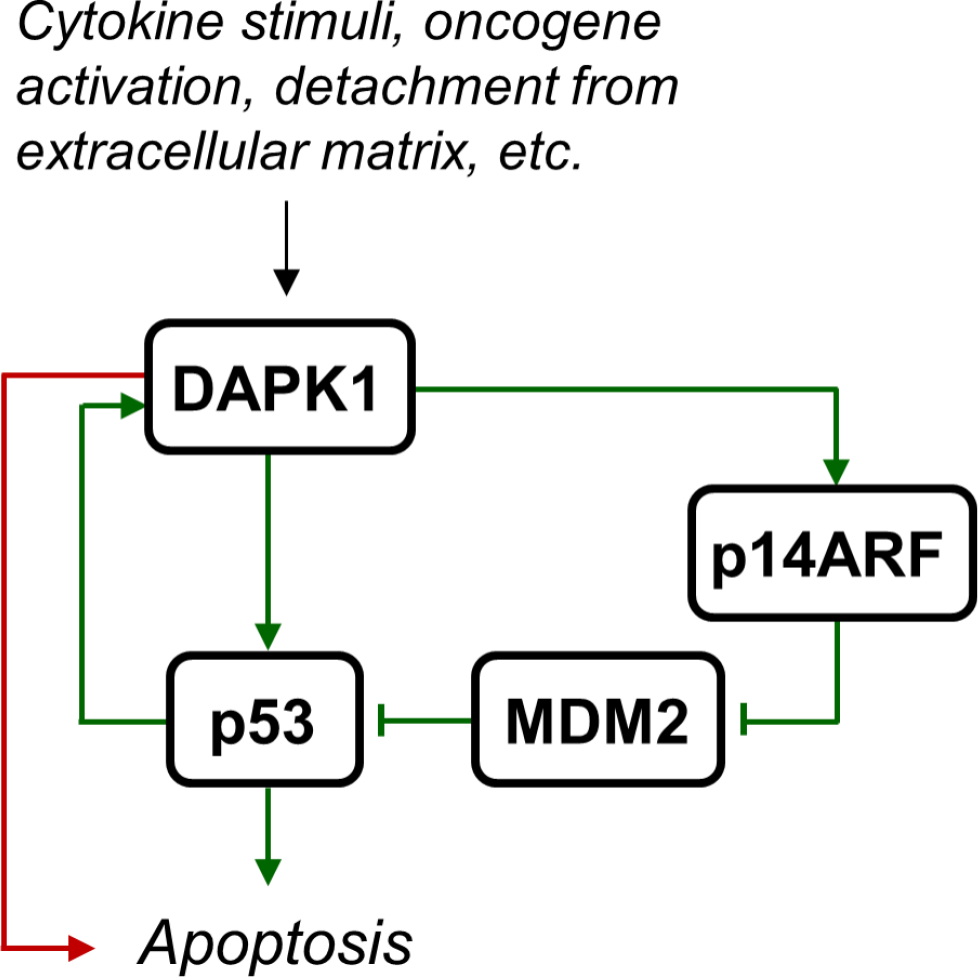
DAPK1 activation leads to apoptosis Various stimuli such as cytokine signalling (e.g. Fas, IFN-γ, and TNF-α), oncogene activation, and detachment from extracellular matrix, may lead to DAPK1 activation, which leads to apoptosis independent of p53 (red arrow) or through direct or indirect activation of p53 (green arrows).

DAP-kinase (DAPK or DAPK1) is a serine/threonine kinase that has a calcium/calmodulin activated autoregulatory domain in its N-terminus. In addition, DAPK1 has a number of extra-catalytic domains, including ankyrin repeats and a death domain, which facilitate interactions with numerous other proteins [3]. Many of these proteins have been implicated in cancer. Most prominent is p53, which is both an indirect and direct substrate of DAPK1. The indirect mechanism of DAPK1 dependent p53-activation is through activation of the ARF tumour suppressor, which inhibits MDM2, an inhibitor of p53. The direct mechanism is through DAPK1 phosphorylation of tetrameric p53 on Ser20, which is located within the transactivation domain that binds p300, leading to p53 activation and apoptosis [4, 5]. In addition, the *DAPK1* gene is a transcriptional target of p53 and, therefore, may be part of a positive feedback loop controlling p53 activation and apoptosis [6]. However, DAPK1 may also facilitate apoptosis independent of p53, and is an essential component in several cell death signalling pathways (Figure 1). Because of its ability to sensitize cells to many of the apoptotic signals that are encountered during malignant transformation *DAPK1* is considered to be a tumour suppressor gene [7].

*DAPK1* has also been shown to be regulated at the transcriptional and translational levels by methylation of its promoter CpG island and by microRNAs, respectively [8]. In several haematological malignancies, including DLBCL, *DAPK1* undergoes DNA methylation-mediated silencing during tumorigenesis. The frequency of *DAPK1* methylation in DLBCL patients is relatively high, but varies somewhat from study to study [9–12]. We have previously shown that almost 90% of DLBCL patients have detectable *DAPK1* methylation [13]. Some controversy exists in the literature whether or not *DAPK1* methylation is a prognostic factor in DLBCL [10–13]. This may be explained by the studied cohorts being small and/or not uniformly treated.

Mutations in the *TP53* gene have been shown to confer a negative effect on survival in DLBCL [14]. Moreover, several studies have shown that *TP53* disruption in combination with other molecular alterations such as deletion of the INK4a/ARF locus at chromosome 9p21 or *MIR34A* promoter methylation, are associated with exceedingly poor prognosis [15–17].

A variety of different methods are available for DNA methylation studies, all having inherent strengths and weaknesses [18, 19]. However, the vast majority does not evaluate allelic methylation patterns. Hence, only very few studies have investigated allelic methylation patterns of tumour suppressor genes in cancer. We, and others, have previously shown that validation of methylation-specific PCR (MSP) products by pyrosequencing provides a sensitive and specific method for the study of methylation [13, 20]. In addition, we designed our *DAPK1* methylation assay to allow allele-specific methylation information to be obtained, as we hypothesized that biallelic methylation of *DAPK1* is a more severe event compared to monoallelic methylation.

In this contribution, we have increased a previously studied cohort [13] to 119 patients uniformly treated with R-CHOP-like regimens and increased the follow-up time. In addition to allelic *DAPK1* methylation patterns, mutation status of the *TP53* gene was evaluated. Potential correlation between *DAPK1* methylation and *TP53* mutations was investigated. Effects on overall survival and disease-specific survival were investigated for *DAPK1* methylation, *DAPK1* allelic methylation patterns, and *TP53* mutations, alone or in combination. Allele-specific expression of *DAPK1* mRNA was studied in a subset of the samples heterozygous for the rs3818584 SNP. In addition, allelic *DAPK1* methylation patterns were studied in a cohort of 67 multiple myeloma patients.

## RESULTS

### *DAPK1* methylation status according to patient characteristics

The clinical characteristics of the DLBCL patients as a function of *DAPK1* methylation status are shown in Table 1. No significant differences in patient or disease characteristics according to *DAPK1* methylation status were observed, with the exception of age, which was borderline significant. This was also the case when analysing clinical characteristics of patients who were methylated and heterozygous for the rs13300553 SNP as a function of monoallelic *DAPK1* methylation of the A-allele (data not shown), with three exceptions: elevated LDH (more patients with monoallelic *DAPK1* methylation of the A-allele had elevated LDH, *p*=0.007), sex (more patients with monoallelic *DAPK1* methylation of the A-allele were male, *p*=0.008), and performance score (more patients with monoallelic *DAPK1* methylation of the A-allele had a poor performance, *p*=0.047). The mean age of the patients with monoallelic *DAPK1* methylation of the A-allele was 62.36 years and 60.42 years for the patients having the other possible allelic methylation patterns (*p*=0.647).

**Table 1:** Clinical characteristics of the DLBCL patients as a function of *DAPK1* methylation status

	Total (n=119)	Unmethylated (n=17)	Methylated (n=102)	*p*-value
**Sex**				
Men	58	8	50	0.88
Women	61	9	52	
**Extranodal involvement**				
Yes	57	8	49	0.94
No	62	9	53	
**Stage**				
I-II	51	8	43	0.71
III-IV	68	9	59	
**Elevated LDH**				
Yes	58	10	48	0.37
No	61	7	54	
**B symptoms**				
Yes	48	5	43	0.32
No	71	12	59	
**IPI score**				
0–2	80	12	68	0.75
3–5	39	5	34	
Performance score^*^				
0–2	113	17	96	0.59
3–5	6	0	6	
**Response**				
CR/Cru	98	16	82	0.36
PD/PR	11	0	11	
Missing/Mors	10	1	9	
**Age at diagnosis**				
Below 65 years	67	11	56	0.45
Above 65 years	52	6	46	
Age, mean (range)	59.8 (22–90)	53.1 years (28–77)	60.7 years (22–90)	0.04

* Eastern Cooperative Oncology Group

LDH, lactate dehydrogenase; IPI, international prognostic index; CR, complete response, unconfirmed complete response; PD, progressive disease; PR, partial response.

### Overall- and disease-specific survival analysis according to *DAPK1* methylation

**Figure 2:**
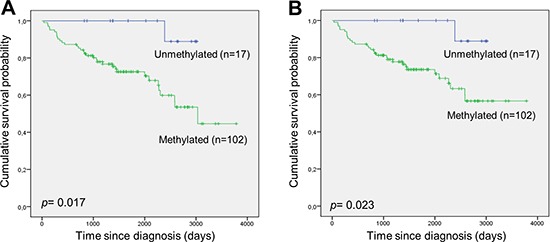
*DAPK1* methylation and survival in DLBCL **(A)** Overall survival according to *DAPK1* methylation. **(B)** Disease-specific survival according to *DAPK1* methylation.

One-hundred and two of the 119 DLBCL patient samples analysed were methylated (85.7%). Only one of the patients having an unmethylated tumour sample died during the follow-up period. Thus, *DAPK1* methylation was associated with shorter overall survival (Figure 2A) and disease-specific survival (Figure 2B). Apparently, this association was not statistically significant in our previous study [13] due to a limited sample size and follow-up time.

### Overall- and disease-specific survival analysis according to allelic *DAPK1* methylation patterns

**Figure 3:**
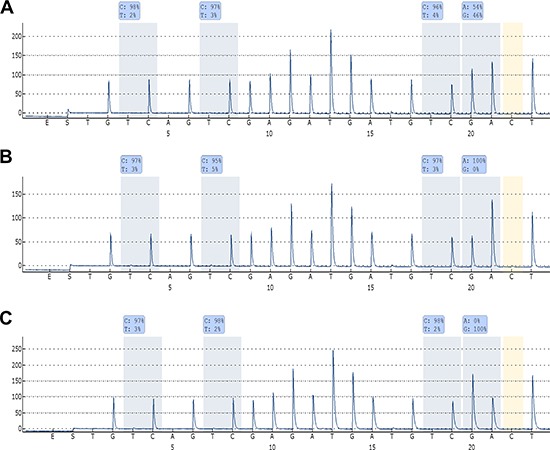
Allelic methylation analyses of *DAPK1* in DLBCL **(A)** Pyrogram of a sample being methylated at both alleles. **(B)** Pyrogram of a sample being methylated only at the A-allele. **(C)** Pyrogram of a sample being methylated only at the G-allele. High methylation levels of the CpG sites indicate that amplification occurred from methylated template, and it can be observed that the bisulfite conversion of the non-CpG C is essentially complete. Thus, these methylation results are regarded as true positive results.

**Figure 4:**
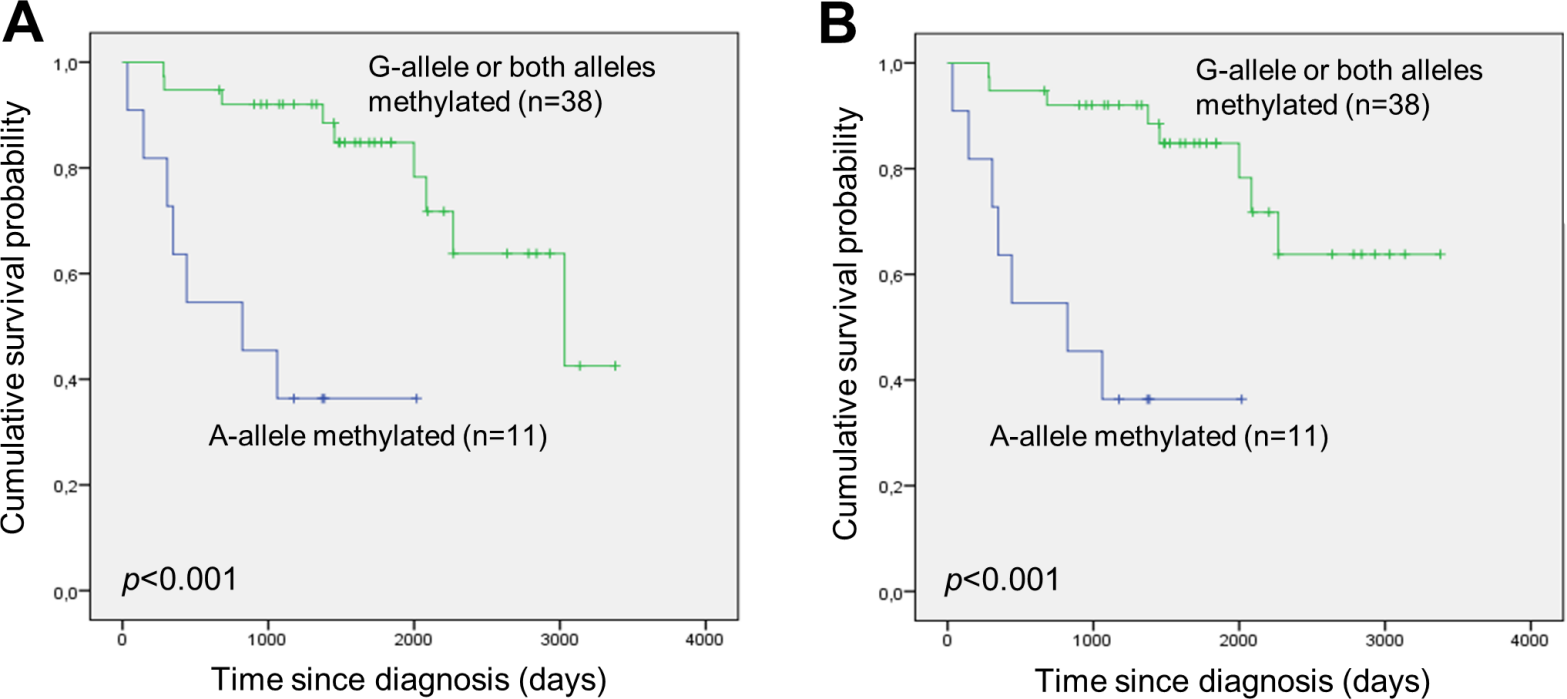
Monoallelic methylation of the A-allele of the rs13300553 *DAPK1* promoter SNP is associated with inferior survival in DLBCL **(A)** Overall survival according to monoallelic methylation of the A-allele and the other possible allelic methylation patterns. **(B)** Disease-specific survival according to monoallelic methylation of the A-allele and the other possible allelic methylation patterns.

Genotyping of the rs13300553 SNP was successful for 109 of the samples. No association was observed between individual genotypes and overall- or disease specific survival in this study (Supplementary Figure 1A and 1B). Fifty-eight of the patients were heterozygous, and pyrosequencing was used to assess their allelic methylation patterns. This was successful for 57 of the samples. Twenty-five were methylated on both alleles, 13 were methylated only on the G-allele, 11 were methylated only on the A-allele, and eight were unmethylated (representative results are shown in Figure 3). It was observed that monoallelic methylation of the A-allele was associated with shorter overall survival (Figure 4C) and disease-specific survival (Figure 4D). There was no difference in survival between patients methylated on the G-allele and patients methylated on both alleles (Supplementary Figure 2A and 2B). In addition, there was no difference in survival between patients being methylated and homozygous AA and patients being methylated and homozygous GG (data not shown).

### Multivariate analysis of disease-specific survival according to *DAPK1* methylation

Univariate analysis revealed that disease-specific survival was also significantly shortened according to the following parameters: stage, elevated LDH, B symptoms, high IPI score, performance score, and age at diagnosis (Table 2). Therefore, these baseline risk factors were included in the Cox proportional hazard model (Table 3). The model identified methylation of *DAPK1* as an independent prognostic factor for predicting disease-specific survival, in addition to elevated LDH. The hazard ratio was larger for *DAPK1* methylation (8.4, 95% CI was 1.13 to 62.88) than for elevated LDH (4.58, 95% CI was 1.78 to 11.83), but the confidence interval was also wider for *DAPK1* methylation.

**Table 2:** Impact of clinicopathological parameters on disease-specific survival in DLBCL

	Total (n=119)	Number of events	*p*-value
**Sex**			
Men	58	20	0.079
Women	61	12	
**Extranodal involvement**			
Yes	57	18	0.27
No	62	14	
**Stage**			
I-II	51	8	0.008
III-IV	68	24	
**Elevated LDH**			
Yes	58	24	<0.001
No	61	8	
**B symptoms**			
Yes	48	18	0.034
No	71	14	
**IPI score**			
0–2	80	14	<0.001
3–5	39	18	
Performance score^*^			
0–2	113	28	0.023
3–5	6	4	
**Age at diagnosis**			
Below 65 years	67	13	0.048
Above 65 years	52	19	

* Eastern Cooperative Oncology Group

LDH, lactate dehydrogenase; IPI, international prognostic index.

**Table 3:** Multivariate Cox regression analyses for prognostic factors affecting disease-specific survival of the DLBCL patients

	Hazard ratio	95% Hazard Ratio Confidence Limits		*p*-value
		Lower	Upper	
**All patients (n=119)**				
Stage (III-IV)	1.971	0.747	5.196	0.170
Elevated LDH	4.584	1.776	11.834	0.002
B symptoms	0.850	0.374	1.930	0.698
IPI score (3–5)	1.156	0.419	3.186	0.779
Performance score (poor)	1.117	0.347	3.601	0.853
*DAPK1* methylation (yes)	8.429	1.130	62.878	0.038
**Methylated and heterozygous patients (n=49)**				
Elevated LDH	7.761	1.514	39.778	0.014
IPI score (3–5)	2.955	0.907	15.185	0.068
Allelic methylation (A-allele)	3.103	0.901	10.691	0.073

### Multivariate analysis of disease-specific survival according to monoallelic methylation of the A-allele

This analysis was performed for the 49 DLBCL patients who were methylated and heterozygous for the rs13300553 SNP. In this patient group univariate analysis revealed that disease-specific survival was significantly shortened in patients with elevated LDH (*p*<0.001) and high IPI score (*p*=0.001). Therefore, these two baseline risk factors were included in the Cox proportional hazard model. In the multivariate analysis only elevated LDH remained as an independent factor predicting disease-specific survival. On the other hand IPI score and monoallelic methylation of the A-allele did not stand out as independent prognostic factors in this patient group (Table 3).

### Allele-specific- and quantitative expression analyses of *DAPK1*

**Figure 5:**
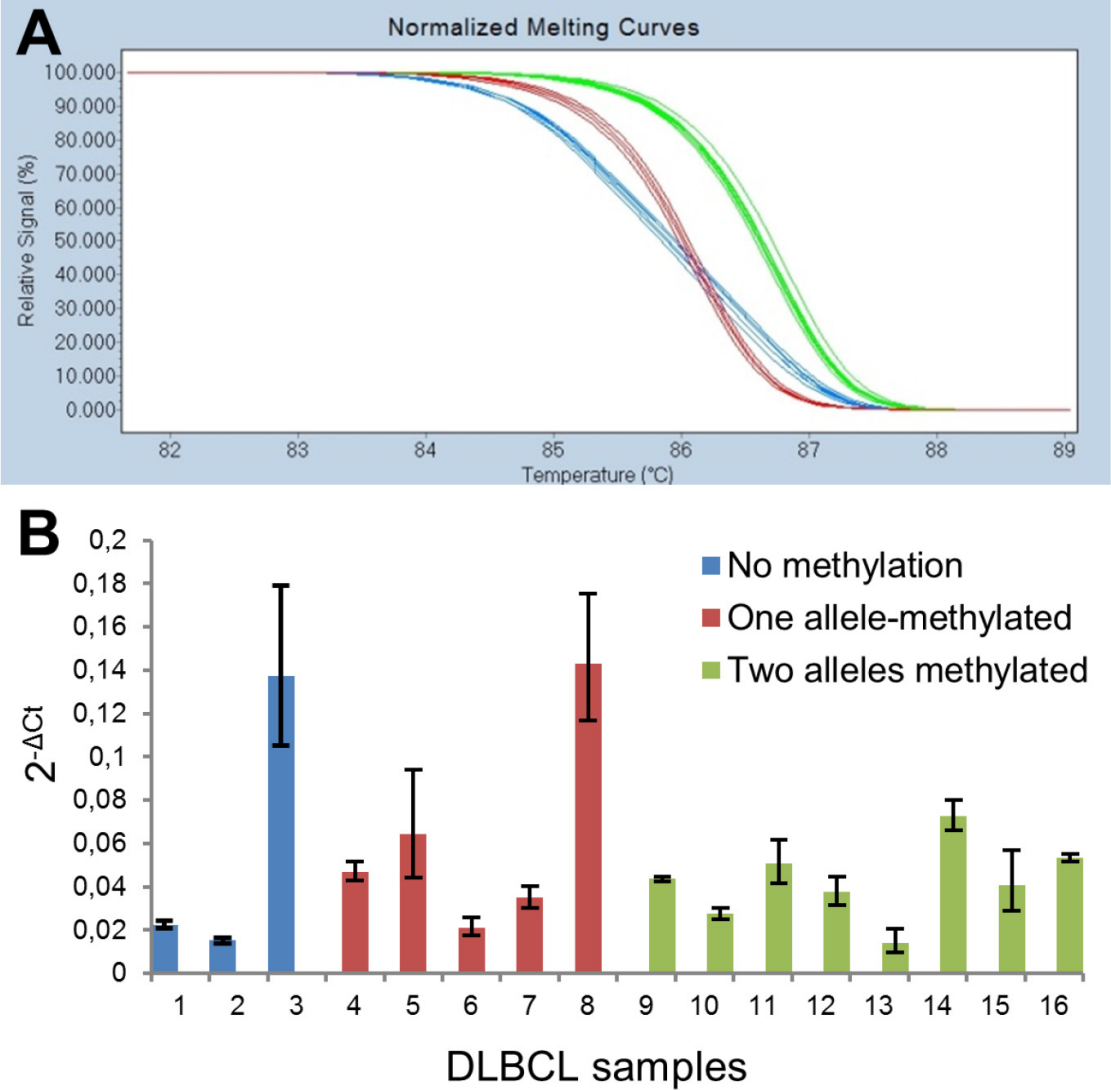
Allele-specific and quantitative expression analyses of *DAPK1* **(A)** HRM analysis of the rs3818584 SNP revealed all 14 heterozygous patients expressed both alleles (blue curves). For the homozygous patients (green and red curves) the HRM analysis was identical to genotyping results. **(B)** Quantitative assessment of *DAPK1* expression in samples for which allelic methylation patterns were analysed. The error bars indicate the highest and lowest possible expression estimates, which can be obtained from the raw Ct-values for *DAPK1* and the reference gene, *PUM1*.

*DAPK1* expression levels and allele-specific expression were assessed for 24 DLBCL samples. Fourteen of these samples were heterozygous for the rs3818584 SNP used to assess allele-specific expression. All fourteen of these samples clearly expressed both alleles as assessed by HRM (representative results are shown in Figure 5A). Eight random samples were selected for confirmation of the HRM results by pyrosequencing, and these analyses gave the same result for all samples (data not shown). Nine of the samples heterozygous for the rs3818584 SNP were also heterozygous for the rs13300553 SNP used to assess allelic methylation patterns, and three of these were methylated only on one allele. Overall, sixteen of the 24 samples were evaluated for allelic methylation patterns (heterozygous for the rs13300553 SNP and/or unmethylated). When assessing the expression levels among these samples no clear picture emerged. *DAPK1* was expressed at very low levels in two unmethylated samples, and at similar levels in samples methylated on both alleles as in samples methylated on one allele, with the exception of one sample (Figure 5B). No RT-enzyme control and no template control were negative.

### Overall- and disease-specific survival analysis according to *DAPK1* methylation and *TP53* mutation status

**Figure 6:**
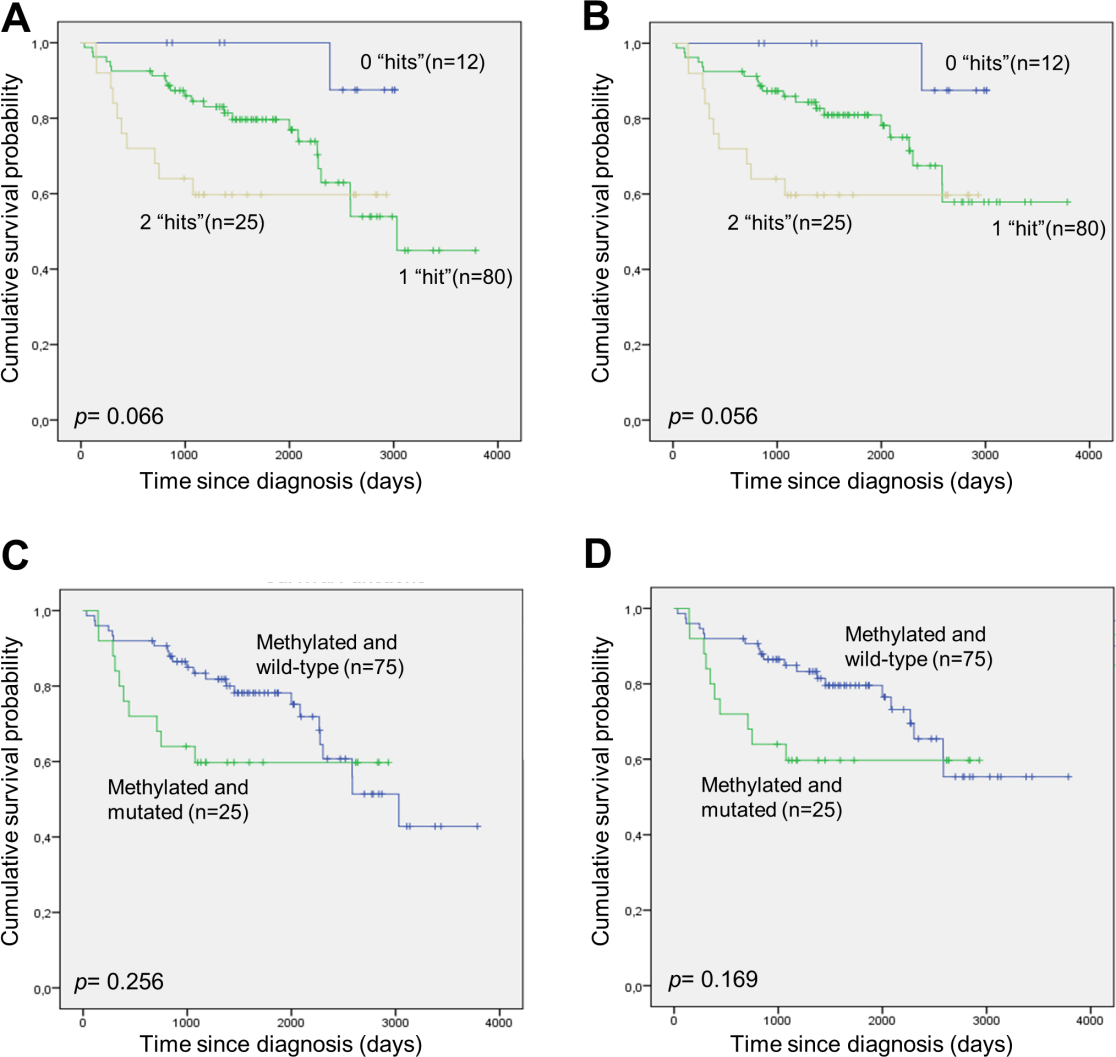
Survival of DLBCL patients with and without *DAPK1* methylation and *TP53* mutation **(A and B)** The patients were divided into three groups, one group without *DAPK1* methylation and without *TP53* mutation (“0 hits”), one group with either *DAPK1* methylation or *TP53* mutation (“1 hit”), and one group with *DAPK1* methylation and *TP53* mutation (“2 hits”). **(A)** Overall survival. **(B)** Disease-specific survival. **(C and D)** The patients were divided into two groups, one group with *DAPK1* methylation and without *TP53* mutation, and one group with *DAPK1* methylation and *TP53* mutation. **(C)** Overall survival. **(D)** Disease-specific survival.

*TP53* mutation status was obtained for 117 of the DLBCL patients (Supplementary Table 1), and 30 were shown to carry a *TP53* mutation (25.6%). Patients with a *TP53* mutation had an inferior overall- and disease-specific survival compared to wild-type patients, however, this was not statistically significant (Supplementary Figure 3A and 3B). Patients carrying both *DAPK1* methylation and a *TP53* mutation had an inferior overall- and disease-specific survival compared to patients carrying only one of these molecular alterations, however, this was borderline statistically significant (Figure 6). Finally, no association between *TP53* mutational status and *DAPK1* methylation was observed (*p*= 0.77).

### Allelic *DAPK1* methylation patterns in multiple myeloma

Fifteen of the 67 multiple myeloma patient samples analysed were methylated (22%). Among the 15 methylated samples six were heterozygous for the rs13300553 SNP. Pyrosequencing was successful for five of these samples, which were all methylated on both alleles (Supplementary Figure 4A). For the remaining sample amplification occurred mainly from the G-allele (80%), however, the pyrosequencing results were uncertain due to low peak heights (Supplementary Figure 4B). Also, the methylation specific PCR resulted in a weak band when performing gel electrophoresis for this sample, indicating that the methylation level was low. Therefore, precise quantification of the methylated alleles was difficult for this sample. No association between overall- and disease-specific survival according to *DAPK1* methylation was observed within this patient cohort (data not shown).

## DISCUSSION

It has been postulated that *DAPK1* methylation is likely to be a prognostic factor that could be used in conjunction with the conventional prognostic factors such as the IPI score [10]. However, another study did not find a correlation between methylation status of *DAPK1* and the prognosis of DLBCL patients [11]. Common for these two studies is that the patient cohorts were relatively small (46 and 53 patients, respectively), and not all patients received treatment with rituximab. We have also previously analysed *DAPK1* methylation patterns in a cohort of 74 uniformly treated DLBCL patients [13]. In this study *DAPK1* methylation was not statistically significantly associated with overall survival despite none of the patients without *DAPK1* methylation died during the follow-up period. Because of these limitations, and because R-CHOP is now standard treatment for patients with DLBCL we increased the cohort to a total of 119 uniformly treated patients with additional follow-up time to investigate whether *DAPK1* methylation can predict overall- and disease-specific survival in DLBCL. Similar to previous studies we used MSP for the detection of *DAPK1* methylation. However, since MSP is prone to false-positive results [20–23], we used pyrosequencing to confirm all positive results.

Our results confirm the potential of *DAPK1* methylation as a prognostic marker in DLBCL treated with R-CHOP, as it was associated with shorter overall survival (*p*=0.017) and disease-specific survival (*p*=0.023). In multivariate analysis *DAPK1* methylation remained an independent prognostic factor for predicting disease-specific survival (*p*=0.038). Furthermore, we observed that, among methylated individuals, monoallelic methylation of the A-allele of the rs13300553 SNP was associated with inferior overall survival (*p*<0.001) and disease-specific survival (*p*<0.001) as compared to the other possible allelic methylation patterns. This is consistent with the finding that monoallelic methylation of the A-allele was associated with elevated LDH (*p*=0.007), suggesting that DLBCL with monoallelic methylation of the A-allele are more aggressive. These observations were surprising as we hypothesized that biallelic methylation would be a more severe event compared to monoallelic methylation.

A possible explanation for this phenomenon could be that the rs13300553 SNP or another genetic alteration acting *in cis*, predispose the allele to become methylated resulting in a constitutional epimutation [24]. This has for instance been observed for the *MSH2* gene in families with Lynch syndrome [25], and, interestingly, in chronic lymphocytic leukemia (CLL) allele-specific expression of *DAPK1* has been shown to occur in non-malignant cells in more than 10% of CLL cases but not in a healthy control population. Moreover, this finding was associated with earlier onset (*p*=0.044) suggesting a potential mechanism for predisposition to CLL [26, 27]. Wei and colleagues also showed that allele-specific expression is correlated with elevated methylation levels in CLL [27], however, it is unlikely that a genetic alteration acting *in cis* predispose the allele to become methylated as no genetic aberrations could be identified in spite of extensive upstream and downstream sequencing [27]. We analysed allele-specific expression of *DAPK1* in 14 DLBCL samples from patients heterozygous for the rs3818584 SNP using HRM analysis of RT-qPCR products containing this SNP. In spite several of these patients carried monoallelic methylation, we observed a robust expression from both alleles in all samples. However, the signal is likely to be derived from normal cells within the tissue sections. Therefore, we believe that germline allele-specific expression is unlikely to play a significant role in DLBCL pathogenesis. Furthermore, we did not observe any associations between allelic methylation patterns and age at the time of diagnosis.

When analysing the genotypes of the rs13300553 SNP it could be observed that, within this patient cohort, it did not influence survival by itself, and it does not affect the amino acid sequence of DAPK1. Therefore, we do not believe that the A-allele by itself influence survival of DLBCL patients.

Another possible explanation could be that the combination of having an A at the position of the rs13300553 SNP in combination with a methylated allele affects the binding of specific proteins and/or nucleosome positioning at the *DAPK1* promoter region. We were, however, not able to study these possibilities as none of the DLBCL cell lines we have screened for allelic *DAPK1* methylation patterns carried monoallelic methylation [13], but we observed that *DAPK1* was expressed at similar levels in samples from patients with monoallelic methylation and patients with biallelic methylation. However, as the tissue sections also contain normal cells, and precise estimation of tumour cell content is difficult [28], it is likely that potential differences remain undetected in a background of normal cells expressing *DAPK1*. In addition, it is likely that the tumour cells are heterogeneous with respect to *DAPK1* methylation and expression, which would also confound the expression analysis. Finally, *DAPK1* may also be downregulated by other mechanisms such as microRNA mediated mRNA destruction.

In the multivariate analysis monoallelic methylation of the A-allele did not remain as an independent prognostic factor for predicting disease-specific survival (*p*=0.07). Also, it was observed that more patients carrying monoallelic methylation of the A-allele were male and had a bad performance score. However, the observed survival differences cannot be solely explained by these factors.

To further investigate a potential role of carrying monoallelic methylation of the A-allele in hematologic cancer, we studied 67 samples from patients with multiple myeloma as *DAPK1* has previously been shown to undergo methylation mediated silencing in this disease [29]. Interestingly, all of the methylated and heterozygous myeloma samples carried biallelic methylation and *DAPK1* methylation was not associated with inferior survival in this patient cohort. Thus, *DAPK1* methylation may play different roles in the tumorigenesis of different haematological malignancies.

*DAPK1* methylation and *TP53* mutation status have not previously been investigated simultaneously in a single cohort of DLBCL patients and associated with survival. In our study, patients carrying both *DAPK1* methylation and a *TP53* mutation had an inferior overall- and disease-specific survival compared to patients carrying only *DAPK1* methylation, however, this was borderline statistically significant. Furthermore, we did not observe a significant correlation between *DAPK1* methylation and *TP53* mutations, and *TP53* mutation status was not significantly associated with overall- or disease-specific survival within this patient cohort. These observations comply with the findings that DAPK1 may facilitate apoptosis both dependent and independent of p53, and two previous studies of *DAPK1* methylation and *TP53* mutations in non-small cell lung cancer also found no associations between these molecular events [30, 31].

In conclusion, we found that *DAPK1* methylation is associated with poor overall- and disease-specific survival in a large cohort of DLBCL patients uniformly treated with rituximab. This finding remained statistically significant in multivariate analyses. Moreover, methylation of the A-allele of the rs13300553 SNP was associated with inferior overall- and disease-specific survival in DLBCL. Germline allele-specific expression of *DAPK1* is unlikely to be responsible for the inferior survival associated with methylation of the A-allele. Finally, DLBCL with combined *TP53* mutation and *DAPK1* methylation is likely to be another “double hit” lymphoma with very poor outcome.

## MATERIALS AND METHODS

### Patient samples, DNA isolation, and bisulfite conversion

This study builds upon two previous studies [13, 15] from which allelic *DAPK1* methylation information and *TP53* mutation status were available for 74 and 62 of the patients, respectively. Additional cases were subsequently collected and analyzed for these two molecular aberrations. The clinical follow-up period for each patient was also prolonged in this study. In total the cohort comprised of 119 DLBCL cases all receiving immunotherapy with rituximab. The diagnoses were based on standard histology and immunophenotyping according to the 2008 WHO classification. The fraction of tumor cells was more than 50% of the total tissue for all samples, and more than 80% for the samples used for expression studies. Cause of death was available for all patients except three. These three patients most likely died from their disease and were analyzed as such. In addition, 67 patients diagnosed with multiple myeloma were included in the present study. From these patients freshly isolated mononuclear cells from the bone marrow were used for isolation of CD138+ plasma cells on a RoboSep (Stem Cell Technologies, Vancouver, Canada). DNA isolation and subsequent bisulfite conversion were performed as described [13].

The project has been approved by the regional ethics committee (De Videnskabsetiske Komitéer, Region Hovedstaden), as a register project according to the Danish ethical regulations, and informed consent was provided for each multiple myeloma patient sample.

### Allelic MSP-pyrosequencing

The *DAPK1* MSP primers were designed to target the sense strand and amplify the region surrounding the rs13300553 SNP (A/G). Several non-CpG cytosines in each of the primers select against the amplification of incompletely converted molecules. Additional CpG sites and non-CpG cytosines in between the primers serve as a control for the amplification of methylated and bisulfite converted template, respectively. An assay specific for unmethylated *DAPK1* sequences was designed to target the same region to verify that negative samples were negative because they were unmethylated and not because the DNA was lost during bisulfite conversion. Genotyping the rs13300553 SNP was done using M-13 tagged *DAPK1* specific PCR primers and sequenced using Sanger sequencing. The primer sequences have been published previously [13]. PCR cycling was performed on the Gene PCR System 9700 (Applied Biosystems). The cycling protocols and concentrations of the reagents used have been published [13]. The PCR amplified DNA was run on 2% agarose gels and sequenced on the PyroMark Q24 (Qiagen) according to the manufacturers' protocol. The same criteria for scoring the samples were applied as in our previous publication [13].

### Detection of *TP53* mutations

The coding sequences and splice sites of exons 5–9 of the *TP53* gene were scanned for mutations by PCR and denaturing gradient gel electrophoresis (DGGE). By covering exon 5–9 it is expected that about 90% of *TP53* mutations will be detected [14]. All mutations were confirmed in a second round of PCR from the original sample, and positive samples were subjected to Sanger sequencing as described [15].

### Allele-specific and quantitative expression analyses of *DAPK1*

RNA was extracted from 24 specimens having at least 80% tumour cells using the miRNeasy mini kit (Qiagen, Hilden, Germany), which extract both microRNAs as well as total RNA, according to the manufacturers' protocol including DNAase treatment. From each sample 250 ng RNA was used for cDNA synthesis, which was performed using the SuperScript^®^ III First-Strand Synthesis System (Life Technologies) according to the manufacturers' protocol. The cDNA was diluted 1:10 before real-time PCR. The LightCycler^®^ 480 instrument II (Roche, Mannheim, Germany) was used for the real-time PCR and high-resolution melting (HRM). The High Resolution Melting Master (Roche) was used at a final concentration of 1X, with 2.5 mM MgCl_2_, 200 nM of each primer, and 5 μL of diluted cDNA resulting in a total volume of 20 μL. The cycling protocol started with one cycle of 95°C for 10 min, followed by 45 cycles of 95°C for 5 s, 60°C for 10 s, and 72°C for 10s, 1 cycle of 95°C for 1 min, 1 cycle of 40°C for 1 min, and a melting step from 65°C to 95°C with 30 acquisitions per °C. All reactions were done in duplicate. The sequences of the *DAPK1* primers were; forward: 5′ Biotin-CAGGGGCTACCACGACAT 3′ and reverse: 5′ AGGGCAATGTGTCCGTCCTT 3′. The reverse primer was designed to overlap an exon-exon junction to prevent amplification of potentially contaminating genomic DNA. The forward primer was biotin labelled to allow confirmation of the HRM data by pyrosequencing, using the PyroMark Q24 (Qiagen) according to the manufacturers' protocol, with the following sequencing primer: 5′ GCTTGCGACAAGGAC 3′. The samples were genotyped for the rs3818584 SNP using the same forward primer as above and reverse: 5′ CCATAAGGCACCTTGTCGCAA 3′ using the PyroMark Q24 (Qiagen) according to the manufacturers' protocol.

For the quantitative analyses the *PUM1* gene was used for normalization as this gene has previously been shown to be stably expressed in lymphoid tissue [32]. The primer sequences were; forward: 5′ CATGCCAGGTTATCCGGTGT 3′ and reverse: 5′ GCGCCTGCATTCACTACAAG 3′. The same concentrations of reagents and LightCycler protocol were used as described above. The data were expressed as relative quantities (2^−ΔCt^) using the average of the Ct-value from each of the duplicates. The highest possible estimate for *DAPK1* expression for each sample was obtained by using the lowest *DAPK1* Ct-value and the highest *PUM1* Ct-value to generate the ΔCt-value, and *vice versa* for the lowest possible estimates.

### Statistical analysis

Statistical analyses were performed in SPSS 19.0 for Windows (SPSS Inc.). Associations between *DAPK1* methylation and *TP53* mutations were assessed using a Fisher's exact test. Correlations between overall survival and disease specific survival and *TP53* mutation status and *DAPK1* methylation status were estimated using the Kaplan-Meier method with the use of a log-rank test. The clinical characteristics and treatment outcomes were compared according to *TP53* mutation status and *DAPK1* methylation status using one-way ANOVA, Person chi-squared tests, or Fisher's exact tests when expected values were below five. For assessment of independent predictors of disease specific survival a multivariate Cox regression hazard model with backward stepwise (likelihood ratio) entry was applied. Effects not meeting a *p*-value< 0.05 in univariate analyses were removed from the model. Any differences were considered to be statistically significant when the *p*-value was <0.05.

## SUPPLEMENTARY FIGURES AND TABLE


