# Supplementary material for: Hypermethylation of DAPK1 is an independent prognostic factor predicting survival in diffuse large B-cell lymphoma
Source: Oncotarget. 2014 Nov 3;5(20):9798–810. doi: 10.18632/oncotarget.2394 (PMC4259438; doi:10.18632/oncotarget.2394)
Supplement: Supplementary file 1 [file oncotarget-05-9798-s001.pdf]

SUPPLEMENTARY FIGURES AND TABLE

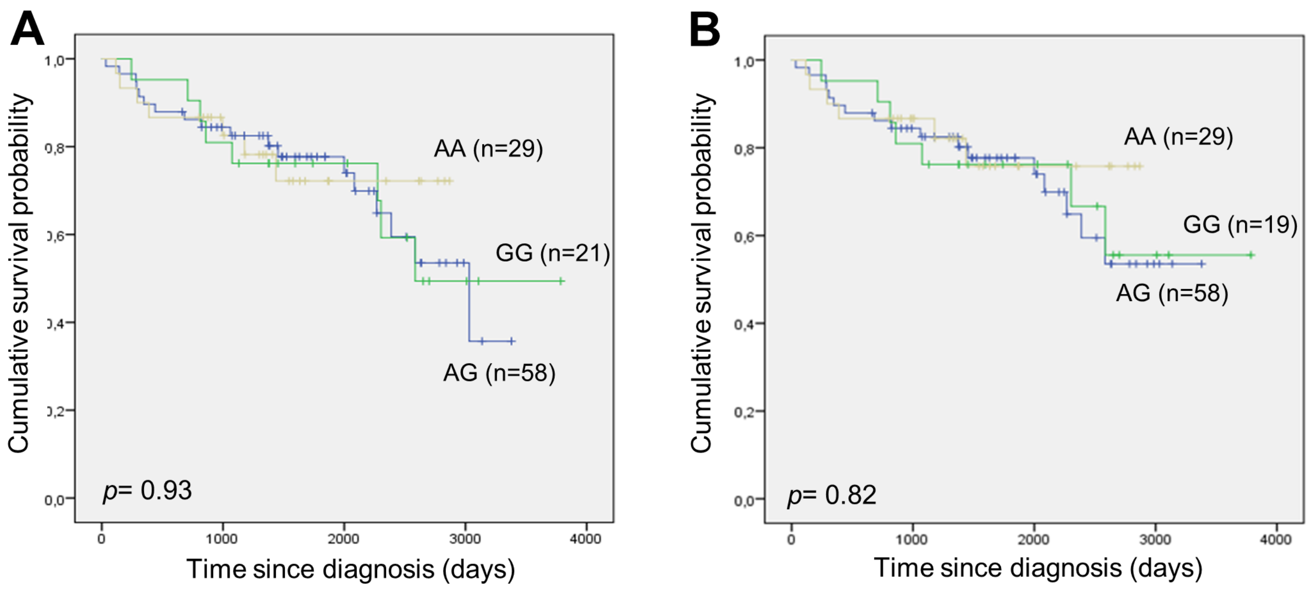

Supplementary Figure S1: Genotypes of the rs13300553 *DAPK1* promoter SNP and survival in DLBCL. (A) Overall survival according to the three different genotypes. **(B)** Disease-specific survival according to the three different genotypes.

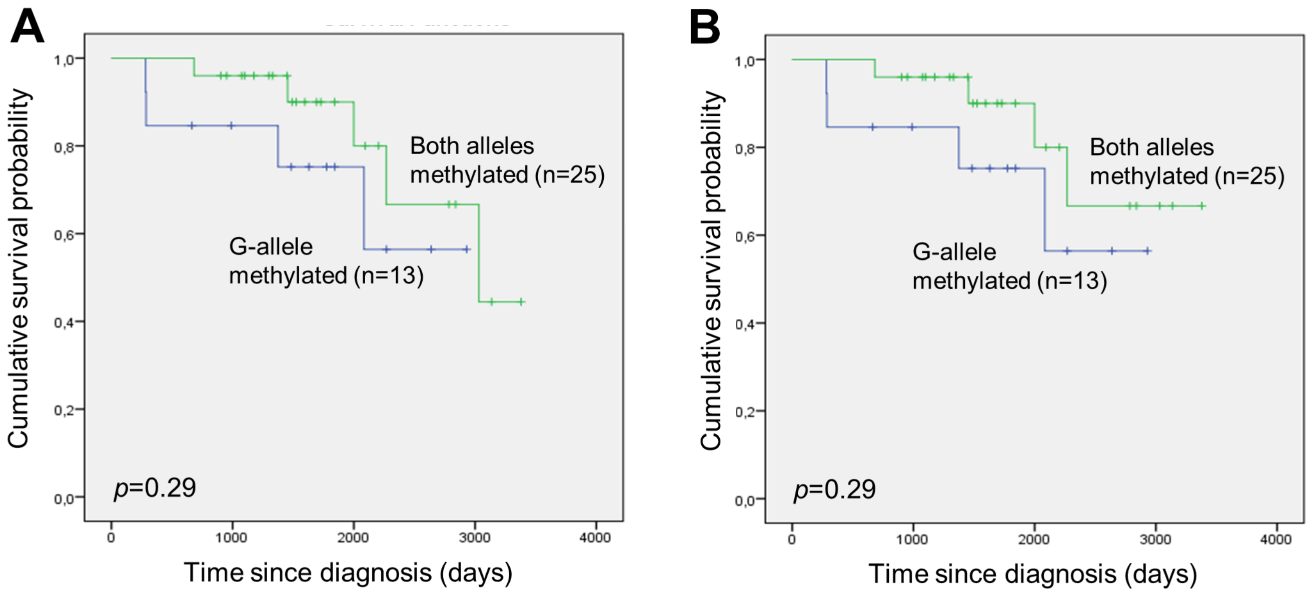

Supplementary Figure S2: No difference in survival between individuals methylated on the G-allele and individuals methylated on both alleles in DLBCL. **(A)** Overall survival. **(B)** Disease-specific survival.

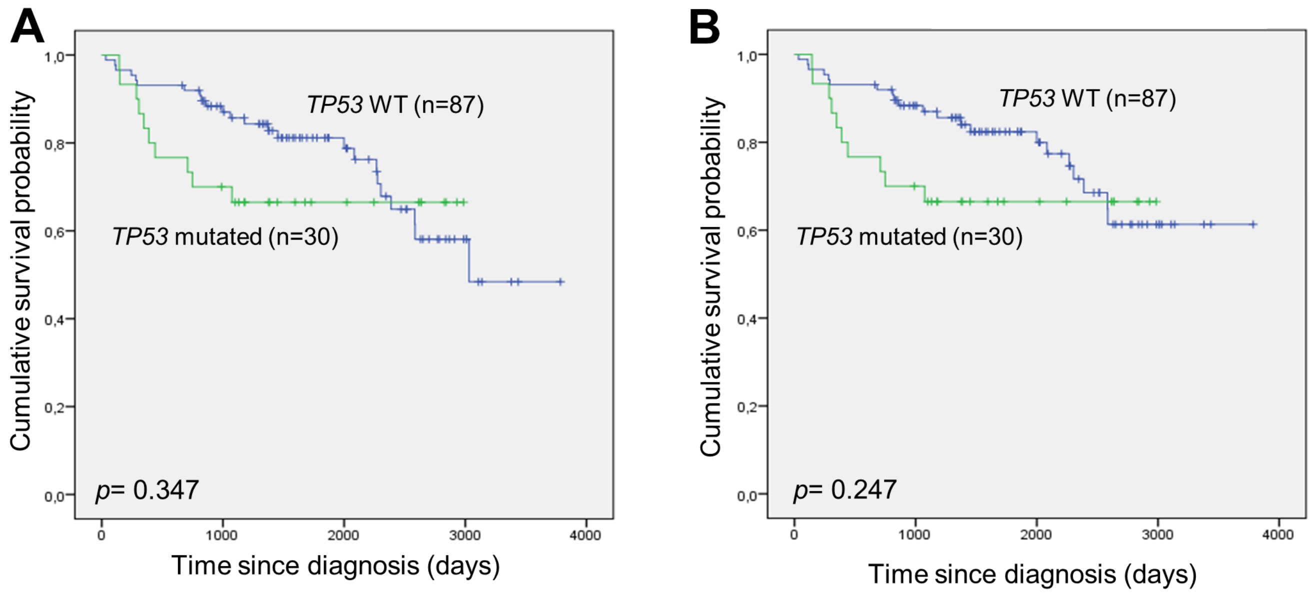

Supplementary Figure S3: Survival of DLBCL patients with and without *TP53* mutation. **(A)** Overall survival. **(B)** Disease-specific survival.

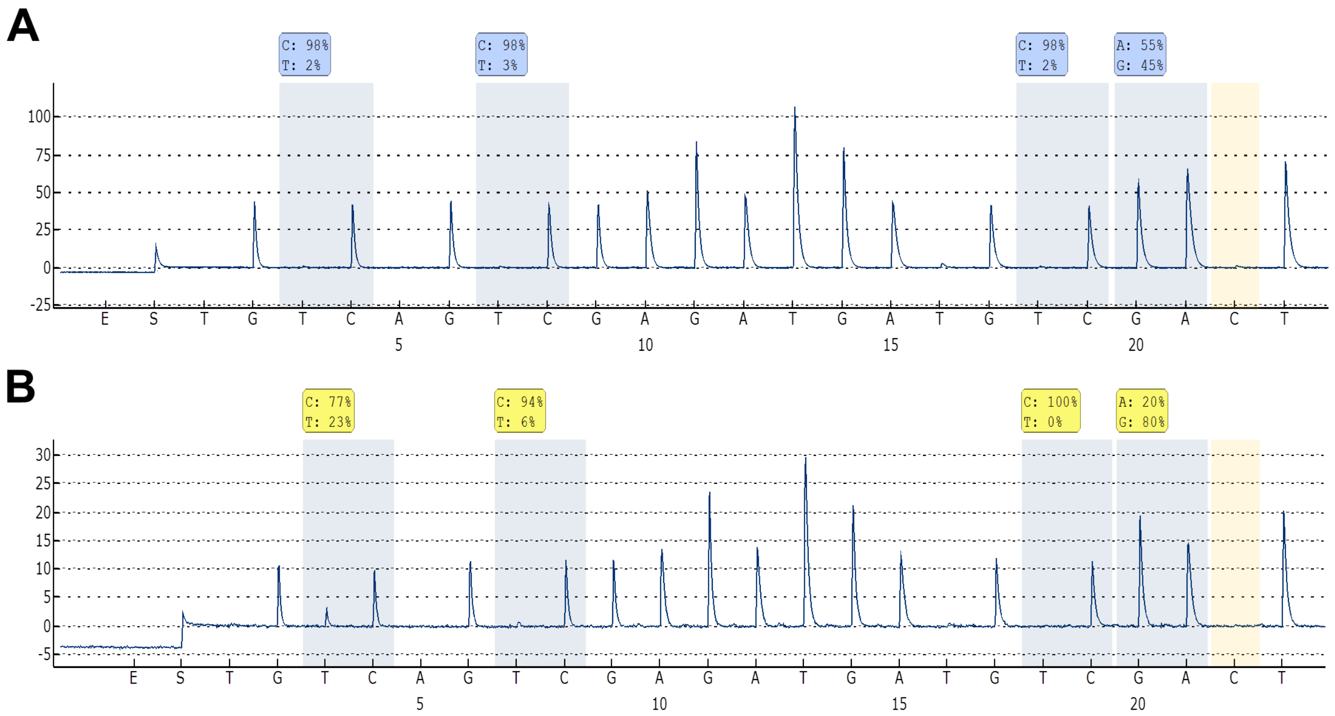

Supplementary Figure S4: Allelic methylation analyses of *DAPK1* in multiple myeloma. (A) Pyrogram of a sample being methylated at both alleles. **(B)** Pyrogram of the sample where amplification occurred mainly from the G-allele (80%). The assessment of allelic methylation patterns in this sample is unreliable, as the pyrosequencing results were uncertain due to low peak heights.

Supplementary Table S1. Overview of the *TP53* mutations detected in 119 DLBCL samples.

Sample ID	Mutation status	Specific mutation(s)
1	no mutation	wild-type*
2	mutation	c521–539 del18bp fs and intron 6+31G>A*
3	mutation	GGC>AGC G244S*
4	no mutation	wild-type*
5	mutation	AAG>GAG;K164E*
6	no mutation	wild-type*
7	mutation	c.559 +1G>A, splice defect*
8	mutation	TGC>TAC; C141Y*
9	no mutation	wild-type*
10	no mutation	wild-type*
11	no mutation	wild-type*
12	no mutation	wild-type*
13	no mutation	wild-type*
14	no mutation	wild-type*
15	no mutation	wild-type*
16	no mutation	wild-type*
17	no mutation	wild-type*
18	no mutation	wild-type*
19	no mutation	wild-type*
20	no mutation	wild-type*
21	mutation	CCC>CTC; P151L*
22	no mutation	wild-type*
23	mutation	c.610_612 ddGAG*
24	mutation	CAC>CAA; H168Q*
25	no mutation	wild-type*
26	no mutation	wild-type*
27	no mutation	wild-type*
28	no mutation	wild-type*
29	no mutation	wild-type*
30	no mutation	wild-type*
31	no mutation	wild-type*
32	no mutation	wild-type*
33	no mutation	wild-type*
34	no mutation	wild-type*
35	mutation	TAC>TGC; Y234C*
36	no mutation	wild-type*

Sample ID	Mutation status	Specific mutation(s)
37	no mutation	wild-type*
38	no mutation	wild-type*
39	no mutation	wild-type*
40	no mutation	wild-type*
41	no mutation	wild-type*
42	mutation	CCC>CTC; P151L*
43	no mutation	wild-type*
44	no mutation	wild-type*
45	no mutation	wild-type*
46	no mutation	wild-type*
47	no mutation	CGA>CGG;R213R*
48	no mutation	CGA>CGG;R213R*
49	no mutation	wild-type*
50	no mutation	wild-type*
51	no mutation	wild-type*
52	no mutation	wild-type*
53	no mutation	wild-type*
54	no mutation	wild-type*
55	no mutation	wild-type*
56	no mutation	wild-type*
57	no mutation	wild-type*
58	no mutation	wild-type*
59	no mutation	wild-type
60	no mutation	wild-type
61	mutation	GGT>AGT; G187S + GTT>ATT; V172I
62	no mutation	wild-type
63	mutation	GTT>ATT; V172I
64	no mutation	wild-type
65	no mutation	wild-type
66	no mutation	wild-type
67	no mutation	wild-type
68	no mutation	wild-type
69	no mutation	wild-type
70	mutation	GTG>ATG; V173M
71	mutation	GAT>AAT; D186N
72	no mutation	wild-type
73	no mutation	wild-type

(*Continued*)

Sample ID	Mutation status	Specific mutation(s)
74	mutation	ACC>ATC; T155I + CCC>TCT; P219S
75	no mutation	wild-type
76	no mutation	wild-type
77	no mutation	wild-type
78	mutation	ATG>GTC; M169V
79	no data	no data
80	no mutation	wild-type
81	no mutation	wild-type
82	no mutation	wild-type
83	no mutation	wild-type
84	mutation	CCC>TCC; P151S
85	mutation	CGG>TGG; R282W
86	no mutation	wild-type
87	mutation	CAA>TAA; Q136stop
88	mutation	CGC>TGC; R156C
89	mutation	CGC>CAC; R175H
90	mutation	GAT>AAT; D186N
91	no mutation	wild-type
92	no mutation	wild-type
93	no mutation	wild-type
94	mutation	CGG>CAG; R248Q and GAA>GTA; E286V
95	mutation	CCT>CTT; P223L
96	no mutation	wild-type
97	mutation	GGC>GAC: G245D
98	mutation	GAT>AAT; D184N
99	no mutation	wild-type
100	no mutation	wild-type
101	no mutation	wild-type
102	no mutation	wild-type
103	mutation	CGT>TGT; R202C
104	no mutation	wild-type
105	mutation	GTG>TTG; V216L
106	no mutation	wild-type
107	no mutation	wild-type
108	mutation	GGC>AGC; G245C + CGC>CAC; R175H
109	no mutation	wild-type
110	no mutation	wild-type

Sample ID	Mutation status	Specific mutation(s)
111	no data	no data
112	no mutation	wild-type
113	no mutation	wild-type
114	no mutation	wild-type
115	no mutation	wild-type
116	no mutation	wild-type
117	no mutation	wild-type
118	no mutation	wild-type
119	mutation	TGC>TAC; C182Y + CCC>TCC; P219S

[*]From previous study (Asmar et al. 2014. Apr 15;5(7):1912–25.)
